# Preoperative and postoperative analgesic techniques in the treatment of patients undergoing transabdominal hysterectomy: a preliminary randomized trial

**DOI:** 10.1186/s12871-015-0046-4

**Published:** 2015-05-06

**Authors:** Jian-qing Chen, Zhen Wu, Lai-you Wen, Jian-zhong Miao, Yong-ming Hu, Ruiping Xue

**Affiliations:** Department of Anesthesiology, The Affiliated Jiangyin Hospital of Nantong University, 163 Shoushan Road, Jiangyin, Jiangsu Province China

**Keywords:** Pre-emptive analgesia, Multimodal, Transabdominal hysterectomy, Stress response, Inflammatory cytokines

## Abstract

**Background:**

Although pre-emptive analgesia is commonly used for the management of postoperative pain in developed countries, no defined protocol has been carried out and widely practiced, especially in transabdominal hysterectomy. Keeping this in mind the present study aimed to investigate the effects of multimodal pre-emptive analgesia on pain management, stress response and inflammatory factors of patients undergoing transabdominal hysterectomy to find an optimized way of pre-emptive analgesia.

**Methods:**

One hundred patients undergoing abdominal hysterectomy were randomly divided into four groups (Trial registration: ChiCTR-IPR-15005848). Group P1 was given intravenous flurbiprofen and epidural fentanyl + ketamine before surgery; Group P2 received intravenous flurbiprofen before surgery and epidural fentanyl + ketamine after surgery; Group P3 was given epidural fentanyl + ketamine before surgery and intravenous flurbiprofen after surgery; Patients in Group C received normal saline treatment.

**Results:**

Compared with control group, the first time to request additional analgesics after surgery were significantly later (P <0.05), 24 h dosage of analgesia were significantly less (P <0.05), VAS score at all time periods after surgery were significantly lower (P <0.05) in Group P1, P2, or P3. At 12 h or 24 h after surgery, VAS score in Group P1 was significantly lower than that in group P2 or P3 (P <0.05, P <0.05). No significant adverse effects were found among the groups (P > 0.05). At 1 or 2 days after surgery, the levels of cortisol, glucose, and IL-6, TNF-α in group P1, P2, and P3 were significantly lower than those in group C (P < 0.05); while, the levels in group P2, P3 were significantly lower than those in group P1 (P < 0.05).

**Conclusion:**

Multimodal pre-emptive analgesia could significantly lower VAS score, inhibit stress response, and reduce inflammatory response in patients undergoing transabdominal hysterectomy, which can be a rational strategy for pain control in future.

**Trial registration:**

ChiCTR-IPR-15005848 on January 17, 2015.

## Background

Transabdominal hysterectomy is an effective strategy for the treatment of benign uterine diseases in primary hospitals, but the operation effect and quality of life were unsatisfactory with large operation trauma, consistent postoperative pain, and adverse postoperative rehabilitation. Besides, for patients undergoing abdominal hysterectomy, stress response and inflammatory reaction harmful for rehabilitation may be also induced by the operation trauma, which manifested as elevated blood pressure, heart rate and blood glucose, and the release of inflammatory factors, such as IL-6 and TNF-α.

Pre-emptive analgesia is a way of pain intervention before noxious stimulation which has been reported to be potent to relieve the postoperative pain by relieving the pain of peripheral and central sensitization [1]. However, the pathogenesis of postoperative pain is complicated involving inflammatory, neurogenic, and visceral mechanisms, and single-mode analgesic medication is not quite efficient [2]. The combination of different analgesic drugs and different methods of analgesia (multimodal pre-emptive analgesia) is a relatively novel method for pain intervention. Though many studies have been reported on multimodal pre-emptive analgesia, no consensus has been achieved, and the effects on stress response and inflammatory reaction related with multimodal pre-emptive analgesia are seldom reported.

Therefore, in the present study, we aimed to find the effects of multimodal pre-emptive analgesia by different combination of preoperative and postoperative analgesic drugs on pain control, stress response and inflammatory factors of patients undergoing transabdominal hysterectomy which may provide reference for clinical analgesia.

## Methods

### Patients

The sample size was determined by using the statistical software program PASS 2008 (NCSS, Kaysville, UT, USA) using preliminary data obtained in our hospital with the following assumptions: α of 0.05 (two-tailed), power of 90%, P1 (Treatment Group Proportion) of 0.7, and P2 (Control Group Proportion) of 0.2. Therefore, a minimum of 18 patients per group was calculated.

One hundred female patients who require transabdominal hysterectomy were enrolled in this randomized and double-blind study from the department of anesthesiology, the Affiliated Jiangyin Hospital of Nantong University, Jiangyin, Jiangsu, China, with approval from the medical Ethics Committee of the Affiliated Jiangyin Hospital of Nantong University and written consent obtained from the participants. The inclusion criteria were physical status American Society of Anesthesiologists (ASA) I- II, aged between 30 and 55 years (mean age of 46.54 ± 8.72), weighted between 50 and 75 kilogram (mean weight of 62.52 ± 12.49 kg) undergoing transabdominal hysterectomy, and the operation time was between 55 and 80 min (mean time of 67.39 ± 12.74 min). Exclusion criteria were contraindication for epidural puncture, known allergy to local anesthetics, the patients with previous history of hypertension, diabetes, coronary heart disease or other medical history, the patients consumed opioids and non-steroidal anti-inflammatory drugs (NSAIDs) preoperatively, and patients with physiological and psychological disorders. A random allocation number table was used for grouping the patients by two experienced chief physician: control group (Group C), multimodal pre-emptive analgesia group 1 (Group P1), multimodal pre-emptive analgesia group 2 (Group P2), and multimodal pre-emptive analgesia group 3 (Group P3). All patients had benign lesions of the uterus, where 83 cases of uterine fibroids, 12 cases of adenomyosis, and 5 cases of endometriosis. No significant difference was found between the groups of patients in age, weight, operation time or history (*P* > 0.05). The participants, care providers, those assessing outcomes were blinded after assignment to interventions.

### Methods

Patients were pre-medicated before anesthesia induction with intramuscular 0.1 mg luminal and 0.5 mg atropine 30 min before operation. After entering the operation room, vital signs of the patients were monitored, peripheral vein was incised, and L_2–3_ lumbar epidural anesthesia was used at the left lateral decubitus position; 3 ml of 0.5% bupivacaine (2 ml of 0.75% bupivacaine and 1 ml of cerebrospinal fluid) was treated at the subarachnoid space; the anesthesia was adjusted below the T8 level; if anesthesia is low, 1.2% lidocaine was treated at the epidural space.

Patients were allocated randomly into one of four groups (25 cases for each group): Patients in Group C (control group) received 5 ml normal saline intravenously before and after surgical operation and 10 ml normal saline epidurally; Patients in group P1 received 50 mg flurbiprofen (Beijing TIDE Pharmaceutical Co.,Ltd, Beijing, China) intravenously and 1.0 μg/kg fentanyl (Yichang Humanwell Pharmaceutical Co.,Ltd, Hubei, China) + 0.25 mg/kg ketamine (Fujian Gutian Pharmaceutical Co.,Ltd, Fujian, China) epidurally before surgical operation, and 5 ml normal saline intravenously and 10 ml normal saline epidurally after surgical operation; Patients in group P2 received 50 mg flurbiprofen intravenously and 10 ml normal saline epidurally before surgical operation, and 5 ml normal saline intravenously and 1.0 μg/kg fentanyl + 0.25 mg/kg ketamine epidurally after surgical operation; Patients in group P3 received 5 ml normal saline intravenously and 1.0 μg/kg fentanyl + 0.25 mg/kg ketamine epidurally before surgical operation, and 50 mg flurbiprofen intravenously and 10 ml normal saline epidurally after surgical operation.

All the patients received transabdominal hysterectomy through traditional abdominal incision in the lower abdomen, and the surgical procedure was similar in all patients, including skin incision, presence and duration of drainage, hospital stay, etc.

Patient-controlled analgesia was used after surgery for all patients: 2 ml/h continuous infusion of 10 μg/kg/100 ml fentanyl (a bolus of 1 ml with a lock-time of 15 min) by using Electronics pump (Shanghai Bochuang Co., Ltd).

We did not make any changes to trial outcomes after the trial commenced, because no significant adverse effects were found in all patients.

### Evaluation indexes

All study–related measurements were taken by the same anesthesiologist who was not aware of the treatment allocation of the patients. The first time to request additional analgesics by pressing the patient-controlled analgesia after surgery was recorded. Pain scores on a visual analogue scale (VAS; 0 = no pain, 10 = worst pain imaginable) were recorded at 6 h, 12 h, 24 h, and 48 h after operation. The 24 h dosage of analgesia and adverse reactions were recorded.

### Outcome measures

Stress response and inflammatory factors of each patient in groups were measured before surgery (8:00 am), 1d postoperative (8:00 am), and 2d postoperative (8:00 am). Stress response was expressed as the concentration of cortisol (Cor) and glucose (Glu). The inflammatory cytokines in serum (IL-6 and TNF-α) were analyzed by commercial ELISA kits (Simens Healthcare Diagnostics Products, Gwynedd, United Kingdom).

### Statistical analysis

The size calculation for each group was as follows: Group C, 24 cases; Group P1, 24 cases; Group P2, 25 cases; Group P3, 25 cases. Statistical analysis was performed by the SPSS16.0 software (SPSS Inc., Chicago, IL, USA). Data were presented as mean ± SD. Patient characteristics (i.e., age, BMI, fasting plasma glucose, HbA1C, and operating time) were analyzed using the independent *t*-test. The differences between the groups were compared by using analysis of variance (ANOVA). The result was considered statistically significant when *P* < 0.05.

## Results

### Patients

**Figure 1:**
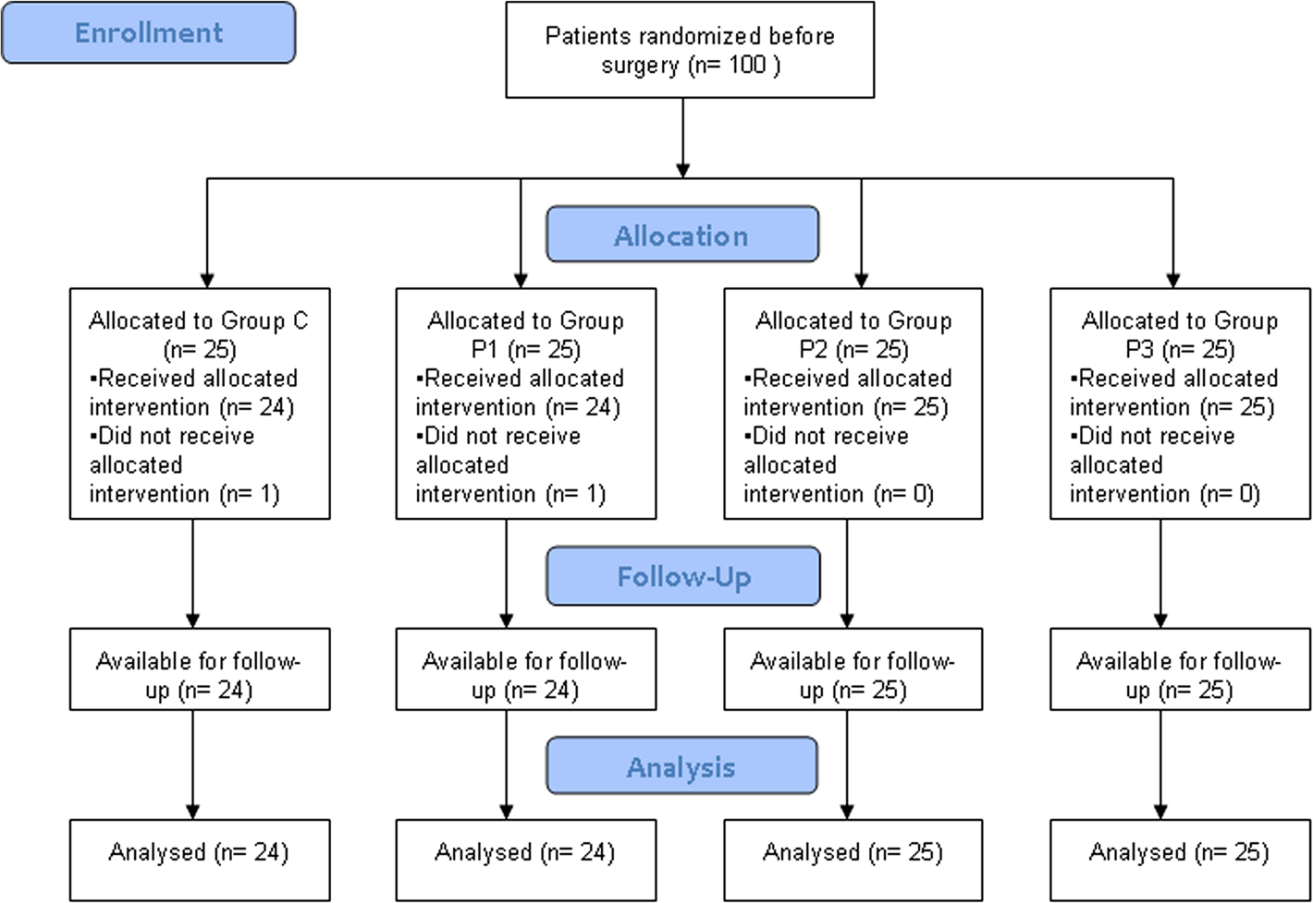
CONSORT flow diagram.

One hundred female patients were enrolled in this randomized and double-blind study from July 2011 to February 2014, and all participants received intended treatment. One patient in group C and one patient in group P1 were excluded for hypopiesia and shock during operation. So data from 98 patients was available for analysis. The CONSORT flow diagram was shown in Figure 1.

The characteristics of participants undergoing transabdominal hysterectomy in four groups were shown in Table 1.

**Table 1:** **Characteristics of participants undergoing transabdominal hysterectomy in four groups**

**Characteristic**	**Group C (n = 24)**	**Group P1 (n = 24)**	**Group P2 (n = 25)**	**Group P3 (n = 25)**
Age (years)	46.72 ± 9.34	45.89 ± 7.82	47.21. ± 8.73	47.42 ± 9.26
BMI (kg/m^2^)	22.64 ± 0.84	23.05 ± 0.76	22.81 ± 0.92	22.74 ± 0.69
Fasting plasma glucose (mmol/l)	5.36 ± 0.48	5.21 ± 0.55	5.42 ± 0.61	5.29 ± 0.39
HbA1C (%)	4.86 ± 0.72	4.83 ± 0.67	4.91 ± 0.58	4.94 ± 0.62
Operating time (min)	66.89 ± 11.72	68.58 ± 13.54	67.51 ± 12.73	68.31 ± 12.86

Data is expressed as mean ± SD. BMI, body mass index. No significant differences among the groups.

### The first time to request additional analgesics after surgery and the 24 h dosage of analgesia

The first time to request additional analgesics after surgery in Group P1, P2 and P3 were significantly later than that in control group (P <0.05), and no significant difference was found among Group P1, P2 and P3 (P >0.05). The 24 h dosage of analgesia in Group P1, P2 and P3 was significantly less than that in Group C (P <0.05), and no significant difference was found among Group P1, P2 and P3 (P > 0.05) (As shown in Table 2).

**Table 2:** **The first time to request additional analgesics after surgery and 24 h dosage of analgesia**

**Groups**	**Cases**	**Initial press time of analgesia (h)**	**24 h dosage of analgesia (ml)**
Group P1	24	8.03 ± 1.56^*^	48.68 ± 1.03^*^
Group P2	24	7.97 ± 1.62^*^	49.12 ± 0.95^*^
Group P3	25	7.99 ± 1.77^*^	49.75 ± 0.73^*^
Group C	25	6.81 ± 1.65	51.01 ± 1.12

Note: *P < 0.05, compared with Group C.

### VAS scores after surgery

At 6 h, 12 h, and 24 h after surgery, VAS score in Group P1, P2, or P3 was significantly lower than that in group C (P <0.05, P <0.05, P <0.05). At 12 h and 24 h after surgery, VAS score in Group P1 was significantly lower than that in group P2 or P3 (P <0.05, P <0.05) (As shown in Table 3).

**Table 3:** **VAS scores at different time period after surgery** $\left(\overline{\mathbf{x}}\pm \mathbf{s}\right)$

**Group**	**Cases**	**6 h after surgery**	**12 h after surgery**	**24 h after surgery**	**48 h after surgery**
Group C	24	2.78 ± 0.85	3.64 ± 0.74	3.79 ± 0.87	3.02 ± 0.71
Group P1	24	2.54 ± 0.81^*^	2.69 ± 0.78^*^&	3.03 ± 0.97^*^&	2.96 ± 0.78
Group P2	25	2.57 ± 0.79^*^	2.79 ± 0.81^*^	3.52 ± 0.84^*^	2.94 ± 0.73
Group P3	25	2.55 ± 0.77^*^	2.83 ± 0.69^*^	3.49 ± 0.79^*^	2.99 ± 0.67

Note: *P < 0.05, compared with Group C. &P <0.05, compared with Group P2 and P3.

### Adverse effect

The patients having nausea after operation were as follows: Group P1, 3 cases; Group P2, 3 cases; Group P3, 5 cases; Group C, 4 cases. No vomiting, pruritus, or psychiatric symptoms reactions were found in all patients of the four groups. No significant adverse effects were found among the four groups (P > 0.05).

### The level of cortisol and glucose in groups

There was no significant difference in serum level of cortisol or glucose between the groups before the surgery (P > 0.05). 1d and 2d postoperative, both the serum level of cortisol and level of glucose in Group P1, P2 and P3 were significantly lower than that in Group C (P < 0.05); and the level of cortisol and level of glucose in Group P2 and P3 were significantly lower than that in Group P1 (P < 0.05) (shown in Table 4).

**Table 4:** **The level of cortisol and glucose in each group before and after surgery**

**Groups**	**Cases**	**Glu (mmol/L)**				**Cor (ng/ml)**	
		**before surgery**	**1d after surgery**	**2d after surgery**	**before surgery**	**1d after surgery**	**2d after surgery**
Group C	24	4.86 ± 0.54	6.89 ± 1.24&	6.28 ± 0.72&	169.54 ± 46.37	235.64 ± 69.57&	206.52 ± 64.37&
Group P1	24	4.83 ± 0.49	6.64 ± 0.62^*^&	6.03 ± 0.69^*^&	165.84 ± 54.36	212.47 ± 57.69^*^&	184.77 ± 60.51^*^&
Group P2	25	4.85 ± 0.59	6.33 ± 0.67^*^	5.84 ± 0.57^*^	172.41 ± 62.38	188.74 ± 64.28^*^	177.58 ± 57.46^*^
Group P3	25	4.81 ± 0.62	6.35 ± 0.59^*^	5.79 ± 0.48^*^	167.58 ± 58.32	191.86 ± 72.58^*^	174.23 ± 67.83^*^

Note: ^*^P <0.05, compared with Group C; &P <0.05, compared with Group P2 and P3.

### The level of inflammatory factors (IL-6 and TNF-α)

There was no significant difference in serum level of IL-6 or TNF-α between the groups before the surgery (P > 0.05). One day or two days after operation, both the serum level of IL-6 and level of TNF-α in Group P1, P2 and P3 were significantly lower than that in Group C (P < 0.05); and the level of IL-6 and level of TNF-α in Group P2 and P3 were significantly lower that that in Group P1 (P < 0.05) (shown in Table 5).

**Table 5:** **The level of IL-6 and TNF-**α **in each group before and after surgery**

**Groups**	**Cases**	**IL-6 (pg/ml)**				**TNF-** **α ** **(ng/ml)**	
		**Before surgery**	**1d after surgery**	**2d after surgery**	**Before surgery**	**1d after surgery**	**2d after surgery**
Group C	24	45.26 ± 5.74	78.48 ± 9.46&	66.38 ± 7.52&	0.88 ± 0.18	1.49 ± 0.16&	1.38 ± 0.17&
Group P1	24	46.35 ± 6.27	71.42 ± 8.79^*^&	61.58 ± 5.82^*^&	0.86 ± 0.13	1.42 ± 0.12*&	1.33 ± 0.13^*^&
Group P2	25	45.81 ± 5.93	65.81 ± 7.68^*^	54.71 ± 6.24^*^	0.87 ± 0.15	1.37 ± 0.13^*^	1.26 ± 0.11^*^
Group P3	25	47.62 ± 7.13	64.98 ± 8.67^*^	55.78 ± 7.62^*^	0.85 ± 0.16	1.36 ± 0.14^*^	1.25 ± 0.12^*^

Note: ^*^P <0.05, compared with Group C; &P <0.05, compared with Group P2 and P3.

## Discussion

Postoperative pain is caused by the association of many factors, including physical injury by surgical incisions, secondary inflammation, visceral pain stimulation, and pain stimulation of nerve endings or central neurons [3]. Therefore, postoperative pain treatment should be combined with comprehensive measures to improve the analgesic effect and reduce adverse reactions. Perioperative pain can cause systemic stress response, and stimulate the release of IL-6, TNF-α and other cytokines involved in inflammation and immune response by mononuclear macrophages and neutrophils. Studies have shown that adequate analgesia can reduce the level of stress response, reduce immune function suppression and promote postoperative rehabilitation.

Pre-emptive analgesia, first introduced by Woolf [4] in 1983, is an antinociceptive treatment that reduces central sensitization and prevents establishment of altered processing of afferent input, which amplifies postoperative pain. Pre-emptive analgesia is found to be potent in preventing severe pain perception which may develop postoperatively [5]. Despite advances in pre-emptive anesthesia and analgesia along with improved delivery systems, single-mode analgesia continues to be the mainstay for treatment of postoperative pain. However, single-mode analgesia such as opioids can oftentimes lead to inadequate pain control or increase in the incidence of adverse events [6]. In the last two decades, evidence-based multimodal analgesia has become increasingly widespread [7]. Multimodal analgesia refers to the combination of different mode of analgesic drugs and incorporation of regional anesthesia to reduce the peripheral and central pain sensitization, and it is a promising alternative that may reduce needs for high doses and dependence on analgesia drugs along with any potential associated adverse effects [2,3]. Therefore, we speculate that the combination of multimodal analgesia and pre-emptive analgesia (multimodal pre-emptive analgesia) may have a better analgesic effect on pain management, reflected by an improved pain scoring, and a reduction of side-effects compared to the morphine group. The aim of this study was to evaluate multimodal pre-emptive analgesia consisting of flurbiprofen, fentanyl and ketamine intravenously or epidurally in patients undergoing transabdominal hysterectomy, and to find the possible mechanisms by exploring the effects on stress response and inflammatory factors.

In this study, intravenous flurbiprofen, and epidural fentanyl with ketamine were used for pre-emptive analgesia study, and patient-controlled analgesia with fentanyl was used after surgery. The results shows that both pre-emptive analgesia with intravenous flurbiprofen, and epidural fentanyl with ketamine can significantly extend initial press time of analgesia, significantly reduce 24 h dosage of analgesia, and significantly reduce 6 h, 12 h, and 24 h VAS scores after surgery, indicating the pre-emptive analgesia effects of different analgesic drugs intravenously or epidurally which is consistent with most reported literatures [8-10]. Flurbiprofen, Fentanyl and Ketamine are three different modes of analgesia drugs. Flurbiprofen, a member of the phenylalkanoic acid derivative family of non-steroidal anti-inflammatory drugs (NSAIDs) used to treat inflammation, has been shown to have pre-emptive analgesia effects by clinical studies [8]. Fentanyl is commonly used as a potent, synthetic opioid analgesic with a rapid onset and short duration of action. Latest studies demonstrated that pre-emptive low dose of fentanyl could effectively prevent fentanyl-induced cough [9,11]. Epidural Fentanyl has also been shown to have pre-emptive analgesia effects. Ketamine is as an *N*-Methyl-D-aspartate (NMDA) receptor antagonist. Sub-anesthetic doses of epidural ketamine can significantly reduce intraoperative and postoperative pain, but 0.15 mg/kg ketamine was reported not efficient in inducing pre-emptive analgesia [12]. In the present study, we find the possible synergistic effect of epidural fentanyl with ketamine in pre-emptive analgesia. Besides, we find significant lower VAS score with multimodul pre-emptive analgesia compared with single mode pre-emptive analgesia, suggesting the possible synergistic effect of multimodal pre-emptive analgesia drugs. We find no significant difference of initial press time of analgesia, 24 h dosage of analgesia, and adverse reactions between multimodal pre-emptive analgesia group and single mode pre-emptive analgesia group, which may be limited to the small amounts of patients, but also suggesting that the safety of intravenous flurbiprofen, epidural fentanyl and ketamine.

Glucose and cortisol are two common indicators widely used for evaluating stress response of body. The higher level of glucose and cortisol indicate the stronger stress response. In the study, we find that 1d and 2d postoperative, both the serum level of cortisol and level of glucose in Group P1, P2 and P3 were significantly lower than that in Group C (P < 0.05) and that in Group P2 and P3 were significantly lower that that in Group P1 (P < 0.05) (Table 3), suggesting that multi-mode pre-emptive analgesia effectively suppress the occurrence of postoperative stress response, and the combination of analgesia drugs with multi-mode mechanisms by different routes of administration shows significant inhibition on stress response, which may be related to the inhibition of pain generation.

IL-6 is an interleukin that acts as a pro-inflammatory cytokine and plays an important role in nerve- endocrine-immune system. IL-6, a functional cytokine with biological activity closely, is associated with immune status, surgical trauma and prognosis. TNF-α is a cytokine with a variety of endogenous biological activity, regulating the inflammatory response and immune response. The elevation of TNF-α is associated with surgical trauma and intensity of pain stimulus. In the study, we found that 1d and 2d postoperative, both the serum level of IL-6 and level of TNF-α in Group P1, P2 and P3 were significantly lower than that in Group C (P < 0.05); and that in Group P2 and P3 were significantly lower that that in Group P1 (P < 0.05) (Table 4), indicating that multi-mode pre-emptive analgesia can effectively suppress postoperative inflammatory reaction in transabdominal hysterectomy, and the combination of analgesia drugs with multi-mode mechanisms by different routes of administration shows better analgesic effect.

Stress response and inflammatory response are always existed before postoperative rehabilitation. The duration of pre-emptive analgesia should include the entire period of noxious stimuli acting on the central nervous system (central sensitization and postoperative incisional pain caused by surgery, and secondary inflammation). However, in the present study, we only observe the effects at 1 day and 2 days after surgery which is a limitation. However, our preliminary study showed that multimodal pre-emptive analgesia was better than a single-mode pre-emptive analgesia without increasing adverse reactions, and multimodal pre-emptive analgesia could significantly lower VAS score, inhibit stress response, and reduce inflammatory response in patients undergoing transabdominal hysterectomy. Though the ideal multimodal pre-emptive analgesic drugs and mode of administration are still not standardized, we believe that with further elucidation in function of the dorsal root ganglia, the development of molecular biology and pain mechanisms, the ideal multimodal pre-emptive analgesia will be an important strategy for pain control clinically.

## Conclusions

The preliminary study showed that multimodal pre-emptive analgesia was better than a single-mode pre-emptive analgesia without increasing adverse reactions, and multimodal pre-emptive analgesia could significantly lower VAS score, inhibit stress response, and reduce inflammatory response in patients undergoing transabdominal hysterectomy, demonstrating that multimodal pre-emptive analgesia could be a rational strategy for pain control. However, the ideal multimodal pre-emptive analgesic drugs and mode of administration are far from standardized which need to be further studied in future.
